# A novel study on the inhibitory effect of marine macroalgal extracts on hyphal growth and biofilm formation of candidemia isolates

**DOI:** 10.1038/s41598-020-66000-1

**Published:** 2020-06-09

**Authors:** Nessma A. El Zawawy, Rania A. El-Shenody, Sameh S. Ali, Mohamed El-Shetehy

**Affiliations:** 1 Botany Department, Faculty of Science, Tanta University, Tanta, 31527 Egypt; 2 Biofuels Institute, School of the Environment and Safety Engineering, Jiangsu University, Zhenjiang, 212013 China; 3 Department of Biology, Faculty of Science and Medicine, University of Fribourg, CH-1700 Fribourg, Switzerland

**Keywords:** Reverse transcription polymerase chain reaction, Antifungal agents

## Abstract

Biofilm formation and hyphal growth are considered to be the most serious virulence factors of *Candida* species in blood causing candidemia infections, which are difficult to treat due to the spread of resistant *Candida* isolates to most antifungal drugs. Therefore, in this study, we investigated the effect of different types and concentrations of selected macroalgal extracts from *Cladostephus spongiosus* (Phaeophyta), *Laurencia papillosa* (Rhodophyta), and *Codium arabicum* (Chlorophyta) in inhibiting those virulence factors of the isolated *Candida*. Acetone extract of *C. spongiosus* (AECS) showed a stronger anticandidal activity against the selected strains than ethanol extract. *Candida krusei* was the highest biofilm producer among the selected isolates. AECS showed an inhibition of *C. krusei* biofilm formation as well as a reduction in the viability of preformed biofilms. Also, AECS reduced various sugars in the candidal exo-polysaccaride layer (EPS). Scanning electron microscopy (SEM) and light microscopic images revealed an absence of hyphae and an alteration in the morphology of biofilm cells when treated with AECS. Moreover, AECS downregulated the expression of hyphal specific genes, hyphal wall protein 1 (*HWP1*), Agglutinin-like protein 1 (*ALS1*) and fourth secreted aspartyl proteinase (*SAP4*), which confirmed the inhibitory effect of AECS on hyphal growth and biofilm formation. Gas chromatography-mass spectrophotometer (GC-MS) analysis of AECS showed three major compounds, which were non-existent in the ethanol extract, and might be responsible for the anticandidal activity; these revealed compounds were 4-hydroxy-4-methyl-2-pentanone, n-hexadecenoic acid, and phenol, 2-methoxy-4-(2-propenyl). These active compounds of AECS may be promising for future pharmaceutical applications in the treatment of candidemia.

## Introduction

*Candida* spp. are one of the most common causes of blood stream infections (candidemia), which were found within hospital patients worldwide^1^. In Egypt alone, the frequency of *Candida* spp. detected in blood samples ranged between 40 to 45% of populations within hospitals^2^. These species produced biofilms that led to high mortality rates^3^. *Candida* spp. possess a number of virulence factors, which enable the organism to cause hematogenous disseminated infections in susceptible hosts^4^. The virulence factors of *Candida* spp. include initial adhesion followed by biofilm production, and morphological transition of yeast cells to their hyphal form^5,6^. The effectiveness of available antifungals in treating candidemia are in decline due to the development of resistant *Candida* biofilms and their toxicity^7–9^. Moreover, azoles that had good broad-spectrum antimicrobial efficacy in candidemia, have many side effects with the prolonged use^10,11^. Thus, there is an urgent need to develop new antifungal compounds for countering *Candida* virulence and pathogenesis.

Inhibition of biofilm production and yeast-hyphal transition are predicted to be effective strategies in the treatment of *Candida* infections when screening for new antifungal agents^12^. Mainly, agents that inhibit biofilm formation and hyphal growth without affecting the viability of planktonic cells, might be useful antibiofilm agents. Past screens have successfully identified compounds from some plant extracts that exhibit antifungal and antibiofilm activities against *C. albicans* such as purpurin, chyrsophanol and rhein^13–16^.

Marine macroalgae are widely employed in folk medicine^17,18^. As well, marine macroalgae have been shown to produce metabolic compounds with antimicrobial^19^, antifungal^20^, anti-inflammatory^21^, antiviral^22^, antioxidant^23^ and anticancer activities^24^. Bioactive molecules of marine algal origin have high potential to inhibit the growth of many bacterial organisms and to further suppress their biofilm metabolic activities^25,26^. Also, El-Sheekh^27^ demonstrated that the extracts of two brown seaweeds, *Sargassum vulgaris* and *Sargassum wightii*, exhibited antimicrobial activities. To the best of our knowledge, there are no studies in which marine algal extracts were investigated as alternatives of anticandidal and antibiofilm agents against candidemia. Therefore, this work aims to evaluate the anticandidal and antibiofilm activities of some seaweed extracts with a preliminary identification of the potential inhibitory compounds to find alternative drugs and a promising source of pharmaceutical agents.

## Results

### Algal extracts showed antifungal activity against the selected *Candida* species

Among different species of algae collected from the Red sea along the coastal region of Hurghada in Egypt, three species, namely, *Cladostephus spongiosus*, *Laurencia papillosa*, and *Codium arabicum*, were evaluated for their potential anticandidal activities. Table 1 revealed that *C. spongiosus* and *L. papillosa* extracts prepared with acetone and ethanol had active compounds that could inhibit growth of the four pathogenic *Candida* selected strains. While, the methanol extract of these algal extracts did not record an anticandidal activity against all the tested *Candida*. Acetone extract of *C. arabicum* showed the lowest anticandidal activity against the selected strains. However, the ethanolic and methanolic fractions of *C. arabicum* did not show any noticeable activity against all organisms. Further from the results obtained, it was observed that all the algal extracts prepared with acetone and ethanol could record higher inhibitory activities against the tested *Candida* compared to fluconazole, which did not show inhibitory activity at the same concentration of extracts (10 µg/ml). Among the algal extracts tested for inhibitory activities, acetone extract of *C. spongiosus* (AECS) showed relatively higher inhibitory activities (20.5, 18.0, 16.7 and 14.7 mm) against the selected *Candida* species (*C. krusei, C. glabrata, C. parapsilosis, and C. albicans*, respectively).

**Table 1:** Algal extracts and susceptibility of *Candida* species.

Algal extract (10 µg/ml)	Solvent	Diameter of inhibition zone (mm)			
		*C. krusei*	*C. glabrata*	*C. parapsilosis*	*C. albicans*
*Cladostephus spongiosus*	Acetone	20.50 ± 0.50	18.00 ± 0.00	16.7 ± 0.29	14.67 ± 0.58
	Ethanol	11.67 ± 0.58	10.67 ± 0.58	5.50 ± 0.50	4.50 ± 0.50
	Methanol	0.00	0.00	0.00	0.00
	**F**	1631.286	2212.00	1824.250	871.000
	***P*-value**	*0.000	*0.000	*0.000	*0.000
*Laurencia papillosa*	Acetone	9.67 ± 0.58	9.17 ± 0.29	9.03 ± 0.06	7.33 ± 0.58
	Ethanol	7.5 ± 0.50	5.17 ± 0.29	5.00 ± 0.00	3.50 ± 0.50
	Methanol	0.00 ± 0.00	0.00 ± 0.00	0.00 ± 0.00	0.00 ± 0.00
	**F**	397.0000	1140.500	55291.00	207.571
	***P*-value**	*0.000	*0.000	*0.000	*0.000
*Codium arabicum*	Acetone	5.33 ± 0.58	2.97 ± 0.06	3.33 ± 0.29	3.33 ± 0.58
	Ethanol	0.00 ± 0.00	0.00 ± 0.00	0.00 ± 0.00	0.00 ± 0.00
	Methanol	0.00 ± 0.00	0.00 ± 0.00	0.00 ± 0.00	0.00 ± 0.00
	**F**	256.000	7921.000	400.000	100.000
	***P*-value**	*0.000	*0.000	*0.000	*0.000
Fluconazole (10 µg/ml)		1.97 ± 0.05	0.00 ± 0.00	1.00 ± 0.29	0.00 ± 0.00

Values are the mean of three replicates ± SD; *significant at P < 0.05

The MIC and MFC values with fungicidal ratios of AECS and fluconazole were shown in Table 2. Both MIC and MFC of AECS gave the lowest value of 80 and 320 µg/ml with a fungicidal ratio of 1:4 in *C. krusei* compared to fluconazole which gave a fungicidal effect at very high concentration of 2000 µg/ml. *C. krusei* was the most susceptible strain to AECS treatment and interestingly, *C. krusei* was found to be the most prolific biofilm producing *Candida* strain (Fig. S2). Herein, we focus on the antifungal activity of AECS on *C. krusei* biofilm production and hyphal growth.

**Table 2:** Minimum inhibitory concentration (MIC) and minimum fungicidal concentration (MFC) of AECS and fluconazole with the corresponding fungicidal ratio.

	Fluconazole		AECS		Isolates	
	Fungicidal ratio	MFC (µg/ml)	MIC (µg/ml)	Fungicidal ratio	MFC (µg/ml)	MIC (µg/ml)
1:5	2000	400	1:4	320	80	*C. krusei*
1:6	2100	350	1:4	360	90	*C. glabrata*
1:4	1200	300	1:4	400	100	*C. parapsilosis*
1:5	1750	350	1:5	450	90	*C. albicans*

### AECS inhibits biofilms formation and eradicates the performed biofilm

**Figure 1:**
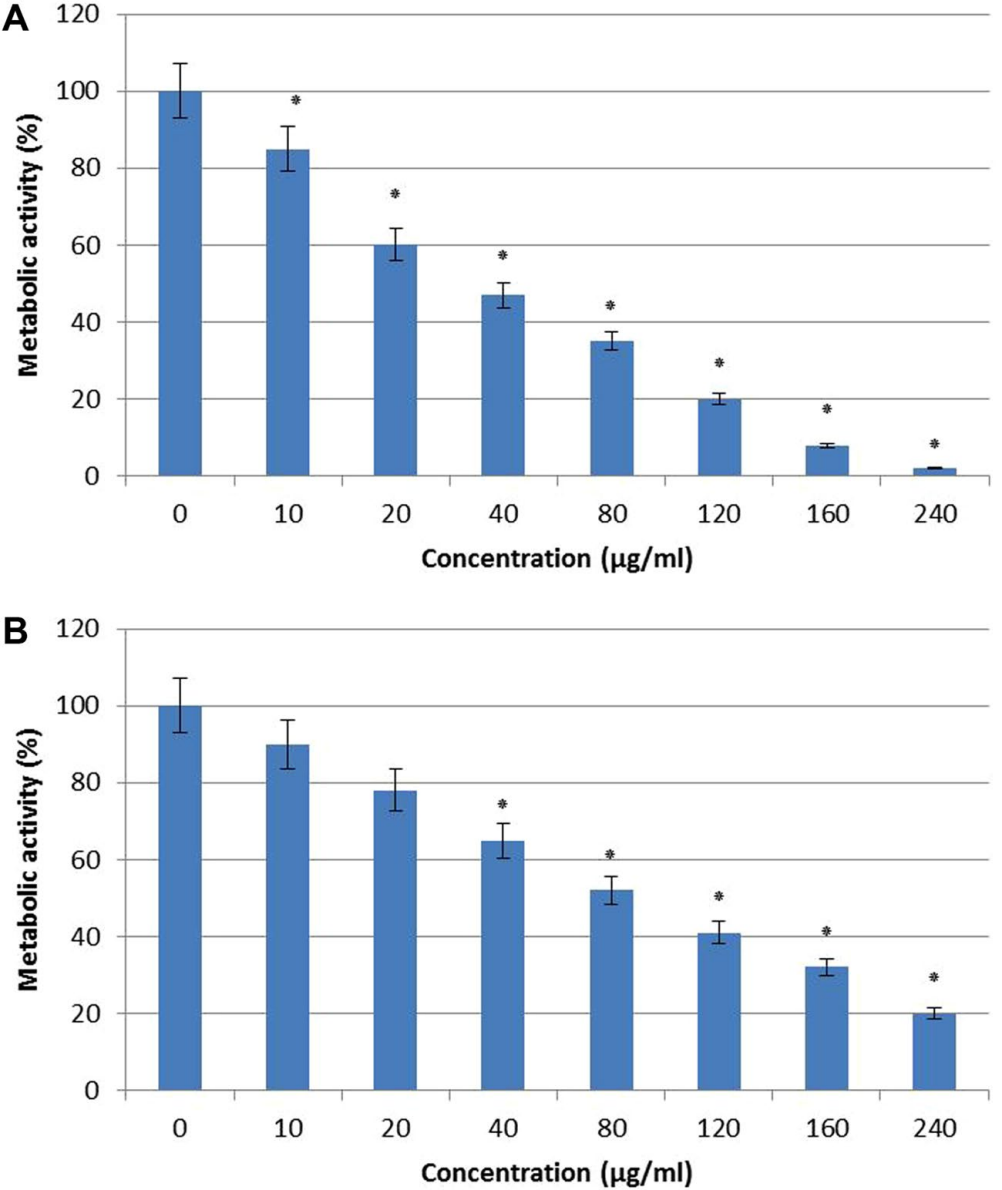
Effect of AECS on *C. krusei* biofilm formation (**A**) and preformed biofilms (**B**) BIC_80_ and BEC_80_ of AECS against biofilm formation and preformed biofilms = 120 and 240 µg/ml respectively. Results represent the average of three independent experiments ±SD. *p < 0.05 when compared with control.

Activity of AECS on *C. krusei* biofilm formation was quantified and viability was expressed in terms of metabolic activity percentage. The Biofilm inhibitory concentration (BIC) of AECS against *C. krusei* was 120 µg/ml (Fig. 1A). The BIC_80_ (biofilm inhibiting concentration) was defined as the lowest concentration of AECS that inhibits 80% metabolic activity of biofilm formation as compared to control. Also, BEC_80_ for *C. krusei* was 2-fold higher (240 μg/ml) compared to (BIC_80_ = 120 μg/ml) (Fig. 1B), as BEC_80_ (biofilm-eradicating concentration) was defined as the lowest concentration of AECS that eradicates 80% of performed biofilm compared to control.

### SEM visualization of *C. krusei* biofilms

**Figure 2:**
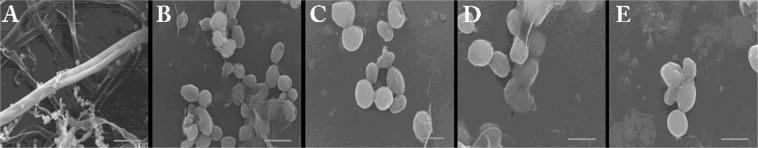
Scanning electron microscope images for the effect of AECS on *C. krusei* biofilm formation at 1000× magnification. 0 μg/ml (**A**), 80 μg/ml (**B**), 120 μg/ml (**C**), 160 μg/ml (**D**) and 240 μg/ml (**E**) of AECS. Scale bar represents 20 μm.

SEM observations provided useful information on the different cellular morphologies present in the biofilm structure. The effect of AECS on *C. krusei* biofilm and its cellular morphology was monitored by SEM (Fig. 2). SEM images of control plates showed the presence of dense complex structure of biofilm having hyphae and yeast cells (Fig. 2A). In the presence of 80 μg/ml AECS, formation of biofilms was reduced with complete hyphal disappearance, and consisted mostly of yeast cells (Fig. 2B). At BIC_80_ of AECS (120 μg/ml), biofilm cells were found to have perforated outer membrane with distorted shape (Fig. 2C). Few yeast cells with wrinkled surface can be seen at 160 μg/ml and 240 μg/ml of AECS concentration respectively (Fig. 2D,E). Further increase in AECS concentration (120 μg/ml) led to a complete inhibition of biofilms.

### AECS inhibits the EPS production and hyphal growth of *C. krusei*

**Figure 3:**
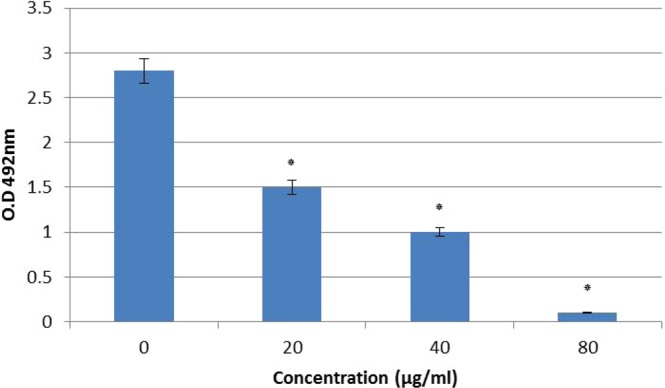
Effect of AECS on the EPS layer of *C. krusei* biofilms. AECS showed a concentration dependent reduction of sugars when compared to that of the control. *p < 0.05.

**Figure 4:**
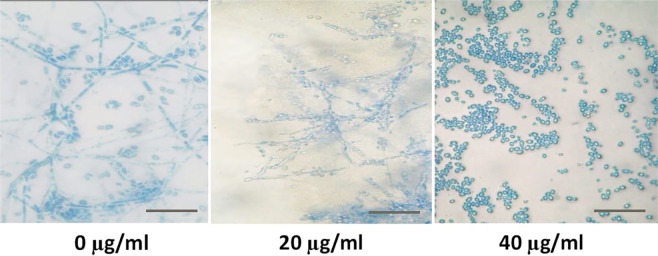
Microscopic visualization for the effect of AECS on *C. krusei* hyphal growth at 40x magnification. Scale bar represents 5 μm.

The major virulence factors of *Candida* species include yeast-to-hyphal transition, and EPS production. EPS ensures the mechanical stability and the physical architecture of the formed biofilms. As a result, we used different concentrations of AECS (20, 40, and 80 µg/ml) to study their effects on the EPS production of *C. krusei*. Our results showed that the different tested concentrations of AECS reduced the amount of sugar content in treated *C. krusei* compared to the control as in Fig. 3 that showed the ability of AECS to decrease sugar content formed by *C. krusei* biofilms. Furthermore, our results showed that AECS inhibits the hyphal growth of *C. krusei* in a dose dependent manner **(**Fig. 4). Microscopically, massive *C. krusei* hyphae were observed in control plates. In the meantime, hyphal growth was moderate at 20 μg/ml, and absent at 40 μg/ml of extract, indicating a directly proportional relation between concentration of AECS and inhibition of hyphal growth.

### AECS downregulates *C. krusei* hyphal specific genes

**Figure 5:**
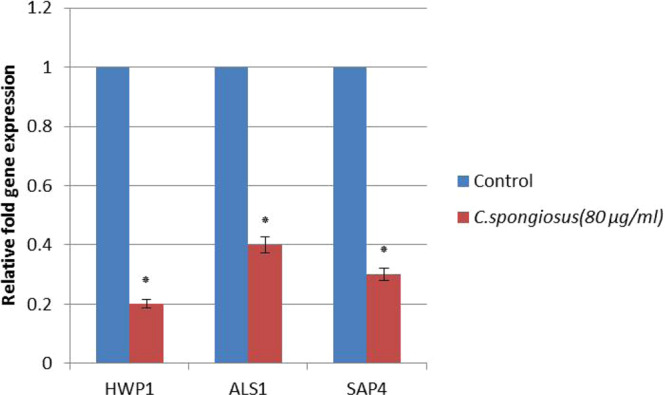
Effect of AECS on the expression of *C. krusei* hypha specific genes. *p < 0.05.

To determine possible molecular mechanism of AECS inhibition of *C. krusei* hyphal growth, we tested the expression level of hyphal growth associated genes such as HWP1, ALS1, and SAP4 genes. Expression of these genes in AECS treated cells was significantly reduced by 5-fold, 2.5-fold, and 3.3-fold, respectively, when compared to the control **(**Fig. 5).

### Chemical analysis of the different *C. spongiosus* extracts using GC-MS

As a next step, it was necessary to check the chemical composition of the different *C. spongiosus* extracts using GC–MS. The chemical constituents, molecular weight and peak area of each component were listed in Tables (3, 4). Our results indicated that the major compounds in AECS were 4-hydroxy-4-methyl-2-pentanone (50.47%), n-hexadecanoic acid (6.46%) and Phenol, 2-methoxy-4-(2-propenyl) (9.21%). While, the three major compounds in the ethanol extract of *C. spongiosus* were 9,12,15-octadecatrienoic acid ethyl ester (22%), Hexadecanoic acid ethyl ester (16.4%), and 2-Hexadecen-1-ol,3,7,11,15-tetramethyl (15.25%). Preliminary screening suggested that these different major compounds in AECS might be the active compounds that cause *C. krusei* biofilm inhibition and hyphal growth. Meanwhile AECS is a mixture of several compounds, each component might contribute to the biofilm inhibition than if they acted alone. So, further study will be done for the isolation and the purification of active compounds with a comprehensive toxicological analysis to determine its safety as it is beyond the scope of this paper.

**Table 3:** Chemical constituents of *C. spongiosus* acetone extract.

S.No	RT	Compound name	PA(%)	Mf	MW
1	8.83	4-hydroxy-4-methyl-2-pentanone	50.47992	C_6_H_12_O_2_	116
2	25.01	Phenol,2-methoxy-4-(2-propenyl)	9.213392	C_10_H_12_O_2_	164
3	27.85	4,7-Octadecadiynoic acid,methyl ester	0.467299	C_19_H_30_O_2_	290
4	29.07	Phenol,2-methoxy-4-(2-propenyl)-acetate	1.582964	C_12_H_14_O_3_	206
5	32.54	Oleic Acid	0.941824	C_18_H_34_O_2_	282
6	32.74	cis-11-Eicosenoic acid	5.487069	C_20_H_38_O_2_	310
7	35.24	Tetradecanoic acid	2.036633	C_14_H_28_O_2_	228
8	36.22	2-Pentadecanone6,10,14-trimethyl	1.12189	C_18_H_36_O	268
9	37.55	Stearic acid,3-(octadecyloxy)propyl ester	0.304654	C_39_H_78_O_3_	594
10	37.87	9-Hexadecenoic acid	0.111443	C_16_H_30_O_2_	254
11	39.66	n-Hexadecanoic acid	16.46548	C_16_H_32_O_2_	256
12	42.94	Oleic acid, eicosyl ester	2.982072	C_38_H_74_O_2_	562
13	46.83	Octadecanoic acid,2-hydroxy-1,3propanediylester	1.726582	C_39_H_76_O_5_	624
14	49.33	Hexa-t-butylselenatrisiletane	2.944481	C_24_H_54_SeSi_3_	506
15	51.60	Cyclodecasiloxane,eicosamethyl	0.423269	C_20_H_60_O_10_Si_10_	740
16	53.57	Decanedioic acid, diisooctyl ester	2.894215	C_26_H_50_O_4_	426

Note: RT-Retention time; MF-Molecular formula; MW-Molecular Weight; PA-Peak area *

**Table 4:** Chemical constituents of *C. spongiosus* ethanol extract.

S.No	RT	Compound name	PA(%)	Mf	MW
1	6.03	Oxime-, methoxy-phenyl	2.265608	C_8_H_9_NO_2_	151
2	9.35	Octadecanal, 2-bromo-	0.800369	C_18_H_35_BrO	346
3	10.89	Propanedioic acid, [2-[(4-methylphenyl) sulfonyl]ethylidene]-, dimethyl ester	5.877589	C_14_H_16_O_6_S	312
4	14.54	9-Octadecenoic yl)methyl ester acid(2-phenyl-1,3-dioxolan-4-	0.308225	C_28_H_44_O_4_	444
5	15.52	Phenol2,4-bis(1,1-dimethylethyl	0.985806	C_14_H_22_O	206
6	16.83	Octasiloxane hexadecamethyl	0.195271	C_16_H_50_O_7_Si_8_	578
7	18.66	Cis-13-Eicosenoic acid	3.9762	C_20_H_38_O_2_	310
8	21.88	2-Hexadecen-1-ol,3,7,11,15-tetramethyl	15.25358	C_20_H_40_O	296
9	22.38	Isopropyl linoleate	2.058717	C_21_H_38_O_2_	322
10	23.24	Docosanoic acid, methyl ester	1.347766	C_23_H_46_O_2_	354
11	24.32	Eicosapentaenoic acid	1.367173	C_20_H_30_O_2_	302
12	24.51	Hexadecadienoic acid, methyl ester	1.257589	C_17_H_30_O_2_	266
13	24.65	6,9,12-Octadecatrienoic acid,, methyl ester	3.249543	C_19_H_32_O_2_	292
14	25.16	Hexadecanoic acid, ethyl ester	16.43712	C_18_H_36_O_2_	284
15	25.27	Hexadecanoic acid	7.717059	C_16_H_32_O_2_	256
16	28.41	Linoleic acid ethyl ester	7.557009	C_20_H_36_O_2_	308
17	28.57	9,12,15-Octadecatrienoic acid, ethyl ester,	22.00678	C_20_H_34_O_2_	306
18	35.65	1,2-Benzenedicarboxylic acid, bis(2-ethylhexyl) ester	3.768015	C_24_H_38_O_4_	390
19	39.43	17-Pentatriacontene	3.441183	C_35_H_70_	490

Note: RT-Retention time; MF-Molecular formula; MW-Molecular Weight; PA-Peak area *

## Discussion

Secondary metabolites of marine algae of potential interest have been extensively documented^25^. According to several reports, antimicrobial activity depends on algal species, extraction method, type of solvent and the resistance of the tested organism^26^. In the present study, acetone was the most effective solvent for the extraction of the bioactive compounds followed by ethanol. Furthermore, *C. spongiosus* was the most effective marine algae against the selected *Candida* species. These results are in agreement with many earlier reports^17,18^. Our data elucidated that AECS showed MIC and MFC at 80 μg /ml and 320 μg /ml against *C. krusei*. These results are consistent with the previous findings of Mickymaray and Allturaiki^28^ who reported that *U. prolifera* demonstrated an MIC and MFC at 500 and 1000 μg/ml against *A. niger*.

Bioactive molecules of marine algal origin have high potentiality to subjugate the growth of many infectious organisms and to suppress their biofilm metabolic activity^26^. Biofilm formation and hyphal morphogenesis are considered the most important virulence factors of *Candida* species^29^. The present study showed that secondary metabolites of AECS have the potential to attenuate these virulence factors.

AECS reduced the metabolic activity of the matured *C. krusei* biofilms *in vitro* and acts as a dominant antibiofilm agent that prevents biofilm formation and removes the existing biofilm. These results agreed with Dulger^30^ who reported that AECS has antibacterial and antibiofilm activities. SEM images of the *C. krusei* biofilm demonstrated the presence of dense hyphae in absence of the extract. However, it showed deformed and swollen cells at BIC_80_. These morphological alterations of the cells resulting in cell death as reported previously for sophorolipid treatment against *C. albicans*^31^. Moreover, cells deformation and distortion of cell membrane have been reported as the mechanisms of antimicrobial activity for many biosurfactants^32^.

AECS suppressed the expression of hyphal genes illustrating the molecular mechanism of AECS in inhibition the hyphal growth. This result is in accordance with Haque^31^ who reported the inhibition of *C. albicans* hyphal growth by sophorolipid using the same genes. To the best of our knowledge, there was not any scientific reports revealing the role of AECS against biofilm formation and hyphal growth of *Candida sp*.

As a next step, it was necessary to check the chemical composition of bioactive secondary metabolites in the different solvent extracts. The differences in the anticandidal effects of the algal extracts may be attributable to differences in the active compounds that present in the algae after their extraction with different solvents. The GC-MS analysis indicated that the chemical composition of the most promising AECS had 3 major peaks in comparison with the ethanolic extract; 4-hydroxy-4-methyl-2-pentanone was the major component, which showed the highest peak area percentage compared with the other components. On the other hand, this compound was not observed in the ethanolic extracts of *C. spongiosus*. Additionally, this compound had previously detected by GC-MS in acetone extract of the red algae *Peterocladia Capillaceae* and *Laurencia pinnatifida* showing a potent antimicrobial activity^33^. As well, this compound was detected as a volatile oil fraction from Phaeophyceae and Rhodophyceae that had an antimicrobial activity^34^. The second major component in AECS was n-hexadecanoic acid, which also was detected using GC-MS from Rhodophyceae^35^, and was reported to have an anticandidal activity^36^. In addition, n-hexadecanoic acid was found as a major component in the acetone extract of *Sargassum hystrix* with a strong antimicrobial activity^33^. Moreover, the third major component observed in AECS was Phenol, 2-methoxy-4-(2-propenyl), which previously identified by GC-MS analysis in methanol extract of *Ulva lactuca* with reported high antimicrobial and antioxidant activities^37^. Collectively, these results suggest that AECS is a mixture of several compounds, and each component might contribute to the biofilm inhibition than if they acted alone. Therefore, the current study suggested that the AECS is a potential source of natural anticandidal agents. It possessed certain metabolites with potent anticandidal properties that may be used for the treatment of blood candidemia infections as it can inhibit the candidal growth by suppressing biofilm formation, hyphal growth and its adhesion genes. Further study is required to characterize the antibiofilm activity of AECS *in vivo* by studying the antagonistic effect of its purified components against *C. krusei* biofilm and its safety.

## Materials and methods

### Organisms and growth conditions

Four *Candida* spp. strains (*C. krusei*, *C. glabrata*, *C. parapsilosis*, and *C. albicans*) were kindly provided by Dr. Mona Osama (Clinical Microbiology Unit, Tanta University Hospital, Faculty of Medicine, Tanta, Egypt). The selected strains were isolated from blood samples collected from the intensive care unit (ICU) and dialysis units in the Tanta University hospital in July 2016. All patients provided written informed consent and the study protocol was approved by the review board of Tanta University Hospitals for the collection of swabs from the Laboratories of Clinical Microbiology Unit at Tanta University Hospital, Faculty of Medicine, Tanta, Egypt. The clinicians followed the guidelines of the Declaration of Helsinki. One strain per patient was studied. Strains were stored at −70 °C. Phenotypic identification was confirmed with the API *Candida* system (bioMérieux Vitek, Hazelwood, MO, USA) following the manual instructions according to standard method of Buchaille^38^. The specific number code for each species is shown in the supplementary data **(**Fig. S1**)**. A frozen glycerol stock of each strain was cultured on sabouraud dextrose broth (SDB; Ifco Laboratories, Detroit, MI, USA) and incubated at 37 °C for 24 h.

### Algal collection

Three seaweeds species, *Cladostephus spongiosus* (Phaeophyta), *Laurencia papillosa* (Rhodophyta) and *Codium arabicum* (Chlorophyta), were collected from Hurghada coastal along the Red Sea (27°15′28″ N; 33°48′42″ E), Egypt, and identified according to Aleem^39^, Abbott and Hollenberg^40^ and Taylor^41^. Collected algal samples were preserved in polythene bags and transferred to the laboratory under cooled conditions to keep temperatures at 4–8 °C.

### Extraction of algal bioactive compounds using organic solvents

About 2 kg of the three isolated algal species were harvested, separately rinsed with sterile-filtered seawater and shade-dried, cut into small pieces, and powdered in a mixer grinder. Then, 5 g of powdered sample of each algal species was extracted separately and soaked with 40 ml of different solvents (acetone, ethanol and methanol) for 48 h. The obtained extracts were filtrated and concentrated in a rotatory evaporator at 40 °C. The residual solvent was removed with a vacuum pump. Then, the weighted crude extracts were well preserved in airtight containers and kept at −20 °C for further analysis^42,43^.

### Anticandidal activity of selected algal extracts

An agar well diffusion method as detailed in El-Zawawy and Hafez^44^ was conducted to determine the most effective algal extract against the four selected strains. Briefly, sabouraud dextrose agar (SDA) plates were inoculated with 100 μl of each *Candida* strain (1 × 10^6^ cells/ml) with wells of size 8 mm filled with 10 µg/ml of each algal extract dissolved in different solvents (acetone, ethanol and methanol). Each solvent (100 µg/ml) was added as a control, which did not show any antifungal activities (data not shown). Fluconazole (10 µg/ml) (Diflucan, Pizer) was used as a positive control. Then, these plates were incubated at 37 °C for 48 h. After the incubation period, the results were observed and the diameter of the inhibition zone around each well was measured to determine the most effective extract. All tests were performed in triplicate.

### Determination of minimum inhibitory concentration (MIC) and minimum fungicidal concentration (MFC)

Minimal inhibitory concentration (MIC) of the most effective extract and fluconazole against the four selected strains was performed using 96-well microtiter plates. Selected strains were added in SDB supplemented with varying concentrations of acetone extract of *C. spongiosus* (AECS) and fluconazole, then incubated at 37 °C for 48 h. After incubation, the fungal growth was assayed at 600 nm using a Biotek plate reader. The MIC was recorded as the lowest concentration that produced complete suppression of visible growth^45^.

The MFC of AECS and fluconazole was determined according to Borman^45^. Briefly, (10 µg/ml) from MIC to last concentration wells of AECS and fluconazole were transferred separately to SDA plates, which were then incubated at 37 °C for 48 h. The MFC was recorded as the lowest drug concentration at which fungal growth was completely inhibited after 48 h of incubation.

### Determining the cell viability of preformed biofilms in *Candida* strains

The ability to obtain quantitatively the metabolic activity of cells in preformed biofilms of the four *Candida* strains were tested by a reduction assay^46^ using colorimetric XTT [2,3-bis (2-methoxy-4-nitro-5sulfophenyl)-2H-tetrazolium-5-carboxanilide sodium salt]. A cell suspension of each *Candida* strain was prepared in SDB at a density of 1 × 10^6^ cells/ml, after that 100 μl were added to each well in microtiter plates. The plates were incubated at 37 °C for 48 h. At the end of incubation, medium was aspirated from the wells and nonadherent cells were removed by washing the biofilms 3-times with a sterile phosphate buffered saline (PBS). Residual PBS of the wells was removed. To each well of prewashed biofilms, 900 μl of fresh broth, 90 μl of XTT salt solution (0.5 mg/ml) and 10 μl menadione solution (1 mM) were added and incubated at dark at 37 °C for 5 h. During incubation, biofilm metabolism reduces XTT tetrazolium salt to XTT formazan. Then, the absorbance was measured spectrophotometrically at 490 nm to obtain the strain which is the higher biofilm producer.

### Effect of AECS on biofilm formation and preformed biofilm of the higher biofilm producer strain

The inhibitory activity of AECS on biofilm formation was assessed *in vitro* according to Ramage^47^. A cell suspension of the selected strain was prepared in SDB (1 × 10^6^ cells/ml) and added to microtiter plates (100 μl per well) with 100 μl of different concentrations of AECS (10, 20, 40, 80, 120, 180, 240 µg/ml). Similarly, 100 μl of SDB with 100 μl of acetone without algal extract were added into wells as a control. Microtiter plates were incubated at 37 °C for 48 h.

Preformed biofilms were prepared as described previously in microtiter plates, then different concentrations of AECS (100 μl) were added into the wells of prewashed biofilms. For the control, 100 μl of SD broth medium with 100 μl of acetone without AECS. Microtiter plates were then incubated, and biofilm metabolic activity was determined as mentioned above by colorimetric XTT assay^46^.

### Biofilm imaging using scanning electron microscopy (SEM)

Untreated and treated biofilms with AECS of selected strains were washed with PBS and air-dried in desiccators^48^. Samples were coated with gold/palladium (40%/60%) and observed in a scanning electron microscope (JEOL, JSM–5200 LV, Tokyo, Japan) at Tanta University, Tanta, Egypt.

### Quantification of exopolysaccharides (EPS) of AECS treated biofilm

This assay was used to estimate the amount of exopolysaccharides in AEC treated preformed biofilm compared to untreated biofilm as a control. Preformed biofilms were prepared as described previously. The non-adherent cells were discarded and 500 µl of 0.9% NaCl was added to the wells of the plate and washed thoroughly. Then, cell suspensions in 0.9% NaCl were transferred to sterile test tubes with an equal volume of 5% phenol. Then, 5% v/v of concentrated sulfuric acid containing 0.2% hydrazine sulfate was added and incubated in dark for 1 h and the absorbance was measured at 490 nm according to Nithya^49^.

### Effect of AECS on candidal hyphal growth

Hyphal growth assay was performed in 10 ml of modified sabouraud glucose broth (MSGB) (Sigma) supplemented with 10% fetal bovine serum (FBS, Invitrogen). A cell suspension of the selected strain (1 × 10^6^ cells/ml) was incubated with different concentrations of AECS (0, 20, 40, 80 μg/ml) at 37 °C with agitation (200 rpm) for 5 h. Aliquots of samples were stained using Lactophenol cotton blue and allowed to dry for 5 min, then visualized under a light microscope using an 40x objective lens and photographed (Nikon Eclipse Ti 100, Japan)^50^.

### Real time PCR (qRT-PCR) expression analysis of candidal hyphal specific genes

Effect of AECS on the expression of hyphal specific genes, hyphal wall protein 1 (*HWP1*), Agglutinin-like protein 1 (*ALS1*) and fourth secreted aspartyl proteinase (*SAP4*), was evaluated by qRT-PCR. Hot phenol/chloroform extraction method^51^ was used in extraction of total RNA from AECS treated (80 μg/ml) and untreated (0 μg/ml) hyphal growth of selected strain. Quantitative RT-PCR amplification mixtures (25 ml) contained 10 ng template cDNA, Light Cycler Hybridization Probes Master Mix kit (Roche diagnostics, Tenay, Turkey), and SYBR Green I master mix buffer with fluorescein. Light Cycler (Roche diagnostics, Tenay, Turkey) and Light Cycler 3.5 software were used^52,53^.

### Gas chromatography-mass spectrometer (GC-MS) analysis

Different extracts from *C. spongiosus* (acetone and ethanol) were investigated for their phytoconstituents using GC-MS (Trace GC Ultra, USA), at the National Research Centre (NRC), El Dokky, Giza Governorate. The identification of unknown compounds was based on comparing their retention time relative to those of the known compounds by matching spectral peaks available with Wiley 9 Mass Spectral Library^54^.

### Statistical analysis

All data were expressed as mean ± standard deviation of three replicates and submitted to variance analysis using SPSS-20.

## Supplementary information

Supplementary information

## Data Availability

The datasets used and analyzed during this study are available from the corresponding author upon request.
