# Supplementary material for: A novel study on the inhibitory effect of marine macroalgal extracts on hyphal growth and biofilm formation of candidemia isolates
Source: Sci Rep. 2020 Jun 9;10:9339. doi: 10.1038/s41598-020-66000-1 (PMC7283248; doi:10.1038/s41598-020-66000-1)
Supplement: Supplementary file 1 — Supplementary information [file 41598_2020_66000_MOESM1_ESM.docx]

**Title page information:**

Type of article:

**Original article**

Title of the article:

**A novel study on the inhibitory effect of marine macroalgal extracts on hyphal growth and biofilm formation of Candidemia isolates**

**Authors**:

**1-Nessma Ahmed El Zawawy**

1. **Rania Abd EL Khalek El Shenody**
2. **Sameh Samir Ali**
3. **Mohamed Hussein El-Shetehy**

**Corresponding author:**

**Nessma Ahmed El Zawawy**

**Email: nesma.elzawawi@science.tanta.edu.eg**

**Telephone: 01289102444**

**Fax:** **(+2) 0403350804**

**Table 1S.Primer list for the targets.**

| Name | Primer sequence |
| --- | --- |
| *Candida* ALS1 Forward | 5’-GAC TAG TGA ACC AAC AAA TAC CAG A-3’ |
| *Candida* ALS1 Reverse | 5’-CCA GAA GAA ACA GCA GGT GA-3’ |
| *Candida* HWP1 Forward | 5’-ATG ACT CCA GCT GGT TC-3’ |
| *Candida* HWP1 Reverse | 5’-TAG ATC AAG AAT GCA GC-3’ |

**
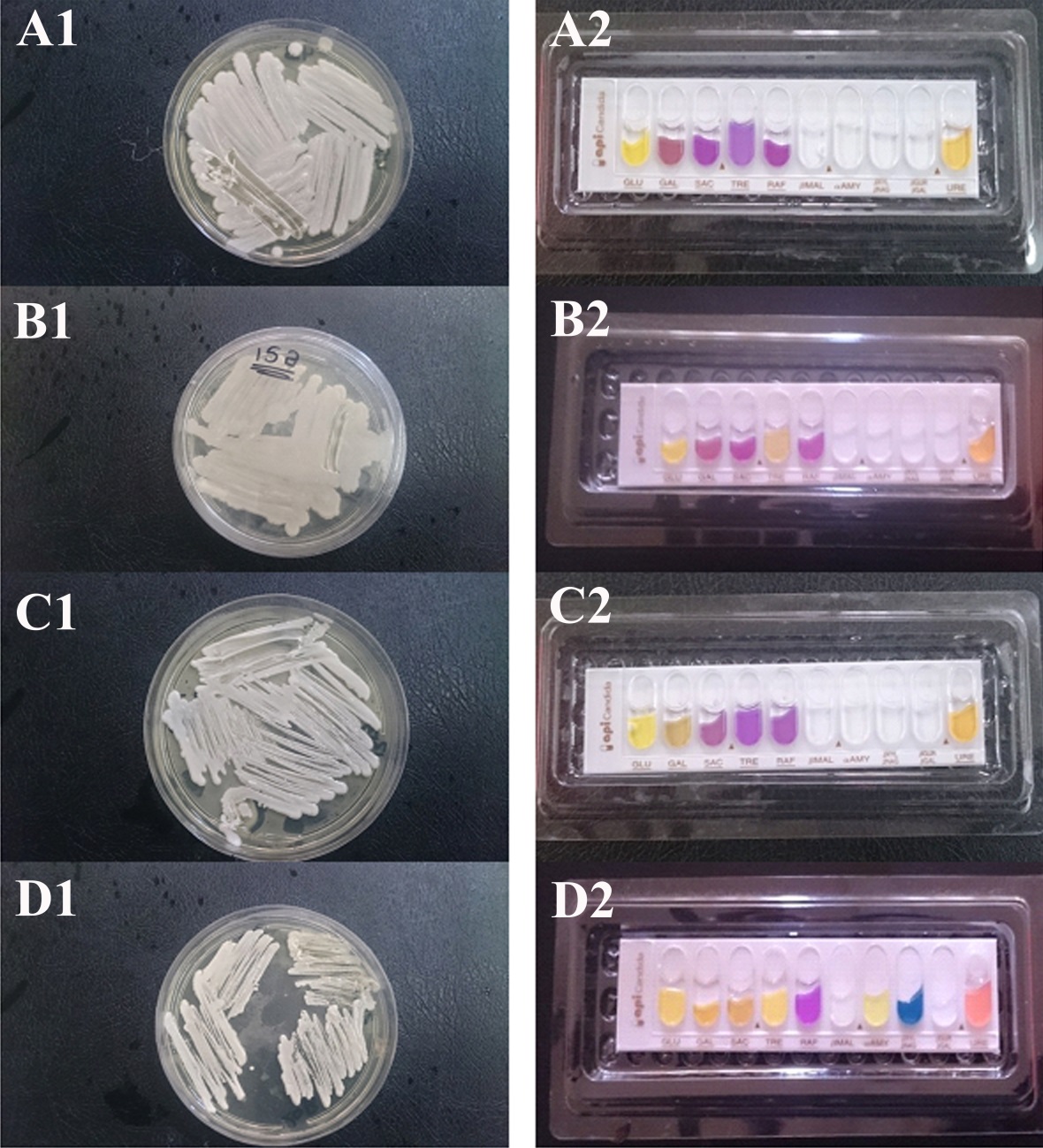
**

**Fig1S. Culture appearance and identification of *Candida* species from blood samples**

٭ Note: A: *C. krusei,* B: *C. glabrata*, C: *C. Parapsilosis*, D: *C. albicans*


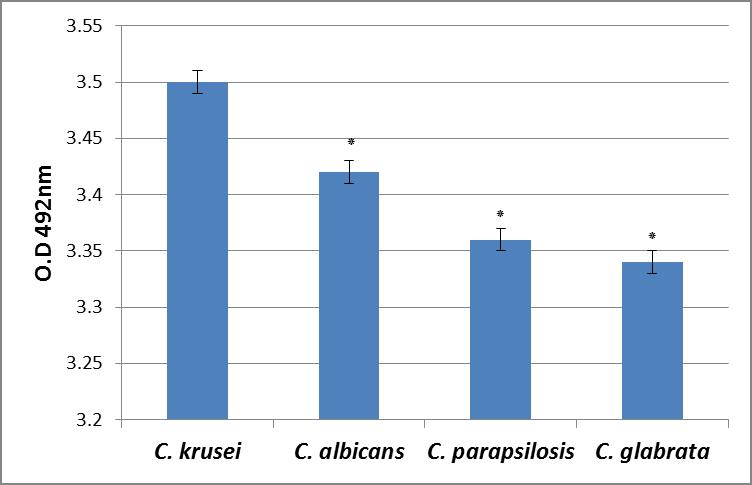


**Fig 2S.**Biofilm formation of *Candida* species.*C. krusei* showed maximum activity.Results represent the average of three independent experiments ±SD. ٭p <0.05.
